# CHILDHOOD DIFFICULTIES AND LATE-LIFE DEPRESSIVE SYMPTOMS: THE ROLE OF EDUCATION AND FAMILY COPING

**DOI:** 10.1093/geroni/igae098.3207

**Published:** 2024-12-31

**Authors:** Seolah Lee, Minju Seong, Miseon Kang, Inhye Jung, Hyo Jung Lee

**Affiliations:** Yonsei University, Seoul, Republic of Korea; Yonsei University, Seoul, Republic of Korea; Yonsei University, Seoul, Republic of Korea; Yonsei University, Seoul, Republic of Korea; Yonsei University, Seoul, Republic of Korea

## Abstract

This study aims to examine how childhood difficulties are associated with late-life psychological well-being. Both adverse experience in origin family, such as family dysfunction and economic hardship, and adulthood economic hardship are known to impede healthy coping and family satisfaction. Thus, using the Cumulative Advantage/Disadvantage Theory, current study further explores how individual’s socio-economic status and family relational aspects in late life mediates the associations between childhood difficulties and depressive symptoms among older Koreans.We use the nationally representative Korean Welfare Panel Study (KoWePS), and select respondents aged 60-79 years old (n = 3,234). A serial mediation model was performed using the Hayes’s PROCESS Macro, bootstrapped 5,000 resamples. The indirect and total effects of childhood difficulties on depressive symptoms in late life were significant. Level of education and coping strategies of family conflict in late life had mediating effects on the relationship between childhood difficulties and older adults’ depressive symptoms. Income in late life, however, hadn’t a mediating effect on the relationship. Family satisfaction in late life had a mediating effect only thorough level of education and coping strategies of family conflict in late life sequentially. Findings suggest that experience of childhood difficulties has an adverse impact on older adults’ psychological well-being through deprivation of education opportunities in childhood and non-constructive coping strategies of family conflict in late life. Tackling inequality in education over one’s life course, as well as helping individuals develop healthy coping strategies, may be considered to mitigate the negative impacts of childhood difficulties on late-life psychological well-being.

